# Age-Stratified Prognostic Value of Cardiopulmonary Exercise Testing Parameters in Patients With Heart Failure

**DOI:** 10.1016/j.jacadv.2026.103008

**Published:** 2026-07-15

**Authors:** Ken Ogura, Kentaro Kamiya, Nobuaki Hamazaki, Emi Maekawa, Chiharu Noda, Momoko Ikeda, Koshi Suzuki, Honoka Mibe, Eishin Konishi, Kohei Matsuda, Kaoru Sato, Takashi Miki, Keisuke Kida, Norio Suzuki, Takeo Fujino, Shoei Yamamoto, Takenori Ikoma, Yusuke Mizuno, Ichiro Matsumoto, Shinjiro Miyazaki, Taisuke Nakade, Yuya Matsue

**Affiliations:** a Department of Rehabilitation Sciences, Graduate School of Medical Sciences, Kitasato University, Sagamihara, Japan; b Department of Rehabilitation, Kitasato University School of Allied Health Sciences, Sagamihara, Japan; c Department of Rehabilitation, Kitasato University Hospital, Sagamihara, Japan; d Department of Cardiovascular Medicine, Kitasato University School of Medicine, Sagamihara, Japan; e Chiharu Heart Clinic, Sagamihara, Japan; f Department of Pharmacology, St. Marianna University School of Medicine, Kawasaki, Japan; g Division of Cardiology, Department of Internal Medicine, St. Marianna University School of Medicine, Kawasaki, Japan; h Department of Cardiovascular Medicine, Kyushu University Faculty of Medical Sciences, Fukuoka, Japan; i Division of Cardiology, Internal Medicine Ⅲ, Hamamatsu University School of Medicine, Hamamatsu, Japan; j Cardiovascular Center, KKR Takamatsu Hospital, Takamatsu, Japan; k Rehabilitation Center, KKR Takamatsu Hospital, Takamatsu, Japan; l Department of Cardiovascular Biology and Medicine, Juntendo University Graduate School of Medicine, Bunkyo, Japan

**Keywords:** cardiopulmonary exercise testing, heart failure, octogenarians, peak VO_2_, VE/VCO_2_ slope

## Abstract

**Background:**

The prognostic value of cardiopulmonary exercise testing (CPET) parameters, particularly peak oxygen consumption (VO_2_) and minute ventilation/carbon dioxide production (VE/VCO_2_) slope, in patients aged ≥80 years with heart failure (HF) remains unclear.

**Objectives:**

The study evaluated the age-stratified prognostic value of CPET parameters for adverse outcomes in patients with HF.

**Methods:**

In this retrospective multicenter cohort study, we included 830 patients with HF who underwent CPET. The primary outcome was a composite of all-cause death or HF hospitalization within 3 years. CPET parameters were examined in 3 age groups: 20 to 64, 65 to 79, and ≥80 years.

**Results:**

During a median follow-up time of 2.8 years, 176/830 patients experienced adverse outcomes. Peak VO_2_ was associated with adverse outcomes in patients 65 years or older (≥80 years: HR: 0.72; 95% CI: 0.62-0.85; 65-79 years: HR: 0.90; 95% CI: 0.84-0.96). VE/VCO_2_ slope was consistently associated across age groups (*P* < 0.05 for all). The area under the curves (95% CI) of peak VO_2_ tended to increase with age (20-64 years: 0.61 [0.53-0.69], 65-79 years: 0.74 [0.67-0.81], ≥80 years: 0.81 [0.70-0.91]), whereas VE/VCO_2_ slope was comparable across age groups (20-64 years: 0.64 [0.56-0.72], 65-79 years: 0.68 [0.60-0.76], ≥80 years: 0.68 [0.55-0.81]).

**Conclusions:**

Although CPET parameters were associated with adverse outcomes across age groups, the prognostic contribution of peak VO_2_ was most pronounced in older patients with HF, highlighting the importance of CPET-based risk stratification in this population.

Cardiopulmonary exercise testing (CPET) is widely used in patients with heart failure (HF) to assess exercise intolerance, guide therapy, and stratify risk.^1^, ^2^, ^3^ Among its parameters, peak oxygen consumption (VO_2_) and minute ventilation/carbon dioxide production (VE/VCO_2_) slope are well-established predictors of mortality and hospitalization.^4^^,^^5^

With increasing life expectancy, the HF population has become markedly older, and patients aged 80 years or older now account for more than one-third of all cases.^6^, ^7^, ^8^ Although exercise capacity generally declines with age,^9^^,^^10^ there is substantial heterogeneity among older adults; chronological age often diverges from biological age, resulting in wide variability in physical reserve and resilience.^11^^,^^12^ Consequently, risk assessment based on chronological age is insufficient, and the evaluation of physiological tolerance is essential for individualized management.

In recent years, advances in pharmacological therapy and a range of less invasive or catheter-based interventions have broadened treatment opportunities for the oldest old patients,^13^, ^14^, ^15^ making precise risk stratification increasingly important. CPET offers a unique opportunity to quantify physiological reserves and predict prognosis, even in this advanced age group. Previous studies have demonstrated the prognostic value of CPET parameters in older patients with HF; however, these studies have included broadly defined older patients (eg, aged ≥65 years, aged ≥70 years),^16^, ^17^, ^18^ with limited representation of octogenarian patients, and have not specifically focused on this age group. As a result, the prognostic value of CPET parameters in patients aged 80 years or older remains insufficiently investigated. Therefore, this study aimed to evaluate the age-stratified associations and predictive capabilities of peak VO_2_ and the VE/VCO_2_ slope for adverse outcomes in HF, with a particular focus on patients aged 80 years or older.

## Methods

### Study design and patient populations

This paper reports the results of a retrospective multicenter cohort study performed at 8 institutions in Japan. We enrolled patients with HF aged ≥20 years at the time of CPET between January 2018 and December 2023 who met the inclusion criteria, with brain natriuretic peptide (BNP)/N-terminal pro-BNP (NT-proBNP) thresholds based on previous study.^19^, ^20^, ^21^

Patients were eligible if they had a history of hospitalization for acute decompensated HF if the BNP >100 pg/mL or NT-proBNP >200 pg/mL in case of sinus rhythm on electrocardiogram at admission, or BNP >120 pg/mL or NT-proBNP >600 pg/mL in case of atrial fibrillation. If a patient with compensated HF had no history of hospitalization, they had to meet the following 2 criteria: 1) in addition to the HF symptoms in the past, blood samples must show if BNP >150 pg/mL or NT-proBNP >300 pg/mL in case of sinus rhythm, or BNP >180 pg/mL or NT-proBNP >900 pg/mL in case of atrial fibrillation; and 2) patients receiving diuretic agents for HF symptoms. The presence of HF symptoms was defined by the following 2 clinical findings: 1) dyspnea induced at rest or with mild exertion; and 2) 2 or more of the following: jugular vein distention, pulmonary rales, leg edema, or pulmonary congestion on chest X-ray.

The exclusion criteria included: 1) previous heart transplantation or the use of an extracorporeal circulation support device; 2) patients on chronic peritoneal dialysis or hemodialysis; 3) patients with acute myocarditis; and 4) patients with missing BNP or NT-proBNP data.

The study protocol was conducted in accordance with the Declaration of Helsinki and was approved by the ethics committee of Juntendo University (E22-0479). Subsequently, it was reviewed and approved at each participating institution. Written informed consent was waived under the Ethical Guidelines for Medical and Biological Research Involving Human Subjects, issued by the Ministry of Education, Culture, Sports, Science, and Technology, the Ministry of Health, Labor, and Welfare, and the Ministry of Economy, Trade, and Industry of Japan. Study information, which included the study objectives, primary outcomes, and eligibility, was published on the University Hospital Information Network (UMIN000050965).

### Data collection

Baseline patient characteristics and medications were obtained at the time of CPET: age, sex, height, weight, body mass index, systolic blood pressure, NYHA functional class, comorbidities (diabetes mellitus, chronic obstructive pulmonary disease), smoking status, and medical therapy (angiotensin-converting enzyme inhibitor or angiotensin receptor blocker, beta-blocker, angiotensin receptor-neprilysin inhibitor, mineralocorticoid receptor antagonist, and sodium glucose cotransporter 2 inhibitor). The following laboratory data obtained within 1 month before or after CPET were used in the study: creatinine, BNP, and NT-proBNP. The value of the left ventricular ejection fraction was also obtained as close as possible to the CPET. CPET parameters, including peak VO_2_, VE/VCO_2_ slope, and peak respiratory exchange ratio (RER), were obtained regardless of whether an ergometer or treadmill was used. In addition, the type of ergometer and the CPET protocol were not restricted.

### Outcomes

The primary outcome was the composite adverse event of all-cause death and HF hospitalization. The time to the endpoint period was calculated as the number of days up to 3 years from the date of the CPET performed to the event’s occurrence. Prognostic data were obtained from medical records, but patients without recurrent presentations to the medical institution or without survival information in the records were regarded as missing data and excluded from the study.

### Statistical analysis

Continuous variables are presented as the mean ± SD or median (IQR), and categorical variables are presented as numbers and percentages. Because BNP showed non-normally distributed data, it was log-transformed. In addition, NT-proBNP was transformed into log-transformed BNP based on a previous study^22^ according to further analyses. The Meta-analysis Global Group in Chronic Heart Failure (MAGGIC) risk score was calculated for each patient based on a previous study.^23^ First, patients were stratified into 3 age groups: ≤64 years, 65 to 79 years, and ≥80 years. Baseline characteristics across age groups were compared using a one-way analysis of variance or the Kruskal-Wallis test for continuous variables and chi-square test for categorical variables. Missing covariate data were handled using multiple imputation and the chained equation method. Results from 20 imputed data sets were combined for survival analyses using Rubin’s formula.^24^ The imputation model included all baseline patient characteristics data collected in this study.

We further divided the patients based on the age-group-specific median value of peak VO_2_ or VE/VCO_2_ slope. Age-stratified Kaplan-Meier curves were constructed and compared using the log-rank test. Adverse events were calculated per 100 person-years to evaluate incidence rates. We also performed age-stratified Cox regression analyses to examine the association of peak VO_2_ or VE/VCO_2_ slope with composite adverse events in unadjusted models and in models adjusted for established risk factors (the MAGGIC risk score and log-transformed BNP). HRs were expressed per increase in each CPET parameter. The proportionality-of-hazards assumption was graphically assessed by Schoenfeld residuals plot. In addition, the interactions between CPET parameters and age for the adverse events were assessed using interaction terms.

Harrell’s C-statistics were calculated to examine the predictive capability and improvement in discrimination of models that consisted of the MAGGIC risk score alone or in combination with log-transformed BNP in the case of adding to peak VO_2_ or VE/VCO_2_ slope. In addition, we analyzed the reclassification capability of adding peak VO_2_ or VE/VCO_2_ slope to the MAGGIC risk score alone or in combination with log-transformed BNP using net reclassification improvement (NRI) and an integrated discrimination index (IDI). These analyses were performed on complete-case analyses, with missing data excluded.

Time-dependent receiver-operating characteristic (ROC) curve analyses of peak VO_2_ or VE/VCO_2_ slope for adverse outcomes were performed by age group, and each area under the curve (AUC) was calculated. In addition, in patients ≥80 years, we determined the optimal cutoff values of each CPET parameter for adverse outcomes using the maximization of the log-rank statistic. The estimated cutoff value’s sensitivity, specificity, positive predictive value (PPV), and negative predictive value (NPV) for adverse outcomes were subsequently calculated.

We also performed 2 sensitivity analyses. First, to confirm the predictive value of peak VO_2_ at maximum effort for adverse events, we only included patients who achieved peak RER ≥1.05, examining them using the same analysis. Second, to address the potential collinearity between age and the age component in the MAGGIC risk score, we constructed a modified MAGGIC risk score excluding the age component and performed Cox regression analyses adjusting for this modified score.

A 2-sided *P* value of <0.05 was considered statistically significant. As this study was intended to exploratorily investigate associations within each age group, the analyses were not adjusted for multiple comparisons. Therefore, the findings should be interpreted as exploratory and hypothesis-generating. Statistical analyses were performed using Stata software (version 18.5; StataCorp LLC) and R (version 4.6.0; R Foundation for Statistical Computing) using R Studio.

## Results

### Baseline patient characteristics

**Figure 1:**
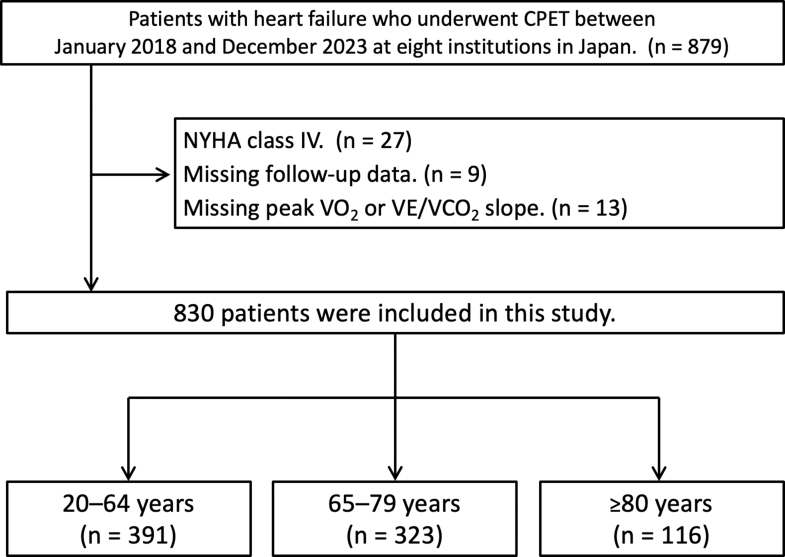
Patients Flow in This Study Of the 879 patients with heart failure who underwent CPET in the study, 830 were included and stratified into 3 age groups. CPET = cardiopulmonary exercise testing; VE/VCO_2_ = minute ventilation/carbon dioxide production; VO_2_ = oxygen consumption.

Of the 879 patients who underwent CPET during the study period, 49 were excluded with NYHA functional class IV, missing follow-up data, or missing peak VO_2_ or VE/VCO_2_ slope; ultimately, 830 patients were included in the analysis (Figure 1). Table 1 shows the patient characteristics and CPET parameters stratified into 3 age groups. Among them, 391/830 patients (47.1%) were aged 20 to 64 years, 323/830 patients (38.9%) were aged 65 to 79 years, and 116/830 patients (14.0%) were aged ≥80 years. Among patients aged ≥80 years, the median age was 83 years (IQR: 81-85), with a full range of 80 to 94 years. The mean peak VO_2_ tended to decline with age (20-64 years: 17.8 ± 6.2 mL/kg/min, 65-79 years: 15.0 ± 4.8 mL/kg/min, ≥80 years: 12.6 ± 3.6 mL/kg/min; *P* < 0.001), whereas the mean of VE/VCO_2_ slope progressively increased with age (20-64 years: 33.9 ± 10.5, 65-79 years: 36.0 ± 9.6, ≥80 years: 39.5 ± 12.0; *P* < 0.001). The mean values of peak VO_2_ by age group are provided in the Supplemental Table 1. Left ventricular ejection fraction was highest in the ≥80 years group compared with the other groups (*P* < 0.001). Concomitantly, the distribution of HF phenotypes differed across age groups, with a higher prevalence of HF with preserved ejection fraction (HFpEF) in the ≥80 years group (63/116 patients, 54.3%) and a higher prevalence of HF with reduced ejection fraction in the 20 to 64 years group (225/391 patients, 57.5%).

**Table 1:** Baseline Patient Characteristics

	Age 20–64 Years (n = 391)	Age 65–79 Years (n = 323)	Age ≥80 Years (n = 116)	*P* Value	Missing Data, n
Age, y	50.0 (42.0-58.0)	72.0 (69.0-76.0)	83.0 (81.0-85.0)	<0.001	0
Male	300 (76.7)	212 (65.6)	79 (68.1)	0.004	0
Height, cm	167.0 (162.0-172.0)	162.0 (154.0-167.1)	157.0 (151.0-163.0)	<0.001	0
Weight, kg	66.6 ± 15.5	58.9 ± 10.9	54.1 ± 9.2	<0.001	0
BMI, kg/m^2^	23.9 ± 4.8	22.6 ± 3.3	21.9 ± 3.0	<0.001	0
Systolic blood pressure, mmHg	105.0 (91.0-121.0)	119.0 (103.0-136.0)	131.0 (113.0-145.0)	<0.001	78
NYHA functional class				0.033	0
I	123 (31.5)	123 (38.1)	52 (44.8)		
II	198 (50.6)	158 (48.9)	45 (38.8)		
III	70 (17.9)	42 (13.0)	19 (16.4)		
LVEF, %	34.7 (22.7-50.3)	47.0 (32.0-60.0)	51.0 (37.0-64.0)	<0.001	4
HF phenotypes				<0.001	4
HFrEF (<40%)	225 (57.5)	122 (37.8)	32 (27.6)		
HFmEF (40%-49%)	64 (16.4)	48 (14.9)	20 (17.2)		
HFpEF (≥50%)	100 (25.6)	152 (47.1)	63 (54.3)		
Comorbidities					
Diabetes mellitus	89 (22.8)	108 (33.4)	37 (31.9)	0.004	0
COPD	10 (2.6)	25 (7.7)	8 (6.9)	0.005	0
Current smoking	81 (20.8)	28 (8.7)	8 (6.9)	<0.001	3
Laboratory data					
Creatinine, mg/dL	1.0 (0.8-1.2)	1.1 (0.8-1.4)	1.1 (0.9-1.5)	<0.001	0
Log-transformed BNP	5.4 (4.4-6.3)	5.7 (5.0-6.3)	5.9 (5.3-6.4)	<0.001	0
Medical therapy					
ACEI/ARB	293 (75.1)	223 (69.0)	73 (62.9)	0.023	1
Beta-blocker	352 (90.3)	266 (82.4)	75 (64.7)	<0.001	1
ARNI	45 (11.5)	22 (6.8)	10 (8.6)	0.093	1
MRA	283 (72.6)	160 (49.5)	40 (34.5)	<0.001	1
SGLT2 inhibitor	93 (23.8)	55 (17.0)	18 (15.5)	0.033	1
CPET parameters					
Peak VO_2_, mL/kg/min	17.8 ± 6.2	15.0 ± 4.8	12.6 ± 3.6	<0.001	0
VE/VCO_2_ slope	33.9 ± 10.5	36.0 ± 9.6	39.5 ± 12.0	<0.001	0
Peak RER	1.2 ± 0.1	1.2 ± 0.1	1.1 ± 0.1	<0.001	0
MAGGIC risk score	15.0 (10.0-20.0)	21.0 (18.0-25.0)	26.5 (24.0-31.0)	<0.001	84
Composite event	67 (17.1)	77 (23.8)	32 (27.6)	0.018	0

Values are mean ± SD, median (IQR), or n (%).

ACEI = angiotensin-converting enzyme inhibitor; ARB = angiotensin receptor blocker; ARNI = angiotensin receptor-neprilysin inhibitor; BMI = body mass index; BNP = brain natriuretic peptide; COPD = chronic obstructive pulmonary disease; CPET = cardiopulmonary exercise testing; HF = heart failure; HFmEF = heart failure with mildly reduced ejection fraction; HFpEF = heart failure with preserved ejection fraction; HFrEF = heart failure with reduced ejection fraction; LVEF = left ventricular ejection fraction; MAGGIC = Meta-analysis Global Group in Chronic Heart Failure; MRA = mineralocorticoid receptor antagonist; RER = respiratory exchange ratio; SGLT2 = sodium glucose cotransporter 2; VE/VCO_2_ = minute ventilation/carbon dioxide production; VO_2_ = oxygen consumption.

### Age-stratified associations of peak VO_2_ and VE/VCO_2_ slope with composite adverse outcomes

**Figure 2:**
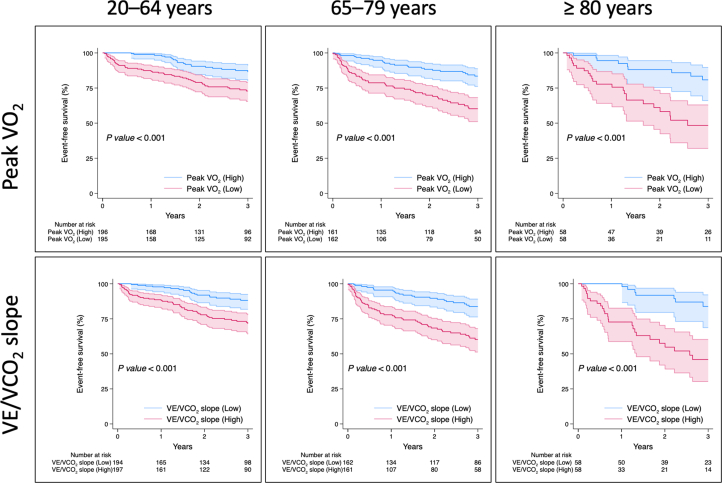
Kaplan-Meier Curves Stratified by Age Groups and Median Peak VO_2_ or VE/VCO_2_ Slope Kaplan-Meier curves show that both peak VO_2_ (top row; high vs low) and VE/VCO_2_ slope (bottom row; low vs high) were significantly different in the incidence of composite events across age groups (20-64 years: left column, 65-79 years: middle column, 80 years or older: right column). Abbreviations as in Figure 1.

Of all patients, those with a complete 3-year follow-up accounted for 369/830 (44.5%) patients. In contrast, 285/830 (34.3%) patients were censored at less than 3 years without an event occurring (20-64 years: 136/391 [34.8%] patients, 65-79 years: 102/323 [31.6%] patients, ≥80 years: 47/116 [40.5%] patients). During a median follow-up of 2.8 (IQR: 1.1-3.0) years, composite adverse events occurred in 176/830 (21.2%) patients (36 all-cause deaths and 140 HF hospitalizations). Event rates were 7.7/100 person-years in the 20–64-year group, 11.5/100 person-years in the 65–79-year group, and 14.6/100 person-years in the ≥80-year group. Estimating survival rates at 3 years for each age group were 79% (IQR: 75-84) in the 20–64-year group, 72% (IQR: 66-77) in the 65–79-year group, and 66% (IQR: 55-75) in the ≥80-year group. Kaplan-Meier survival analyses showed that low peak VO_2_ or a high VE/VCO_2_ slope had higher composite event rates than comparison patients across age groups (log-rank *P* < 0.001 for all) (Figure 2). Table 2 shows the results of univariate and multivariate Cox regression analyses of composite events based on continuous peak VO_2_ and VE/VCO_2_ slope. In multivariate analysis adjusted for the MAGGIC risk score and log-transformed BNP, patients aged ≥65 years with higher peak VO_2_ had lower HR (65-79 years: adjusted HR: 0.90; 95% CI: 0.84-0.96; ≥80 years: adjusted HR: 0.72; 95% CI: 0.62-0.85); however, there was no significant association with composite events in those under 65 years. Moreover, interactions were presented between peak VO_2_ and age (*P* < 0.05 for all). Conversely, a higher VE/VCO_2_ slope consistently showed significantly higher HR across the age groups, even after multivariable adjustment. In addition, no interactions were found between VE/VCO_2_ slope and age.

**Table 2:** Cox Regression Analyses for Peak VO_2_ and VE/VCO_2_ Slope as Predictors

	Age 20–64 Years HR (95% CI)	Age 65–79 Years HR (95% CI)	Age ≥80 years HR (95% CI)	*P* for interaction
Peak VO_2_				
Unadjusted	0.92 (0.88-0.97)^a^	0.85 (0.80-0.90)^a^	0.70 (0.60-0.82)^a^	0.001
MAGGIC risk score	0.96 (0.91-1.01)	0.88 (0.83-0.94)^a^	0.72 (0.62-0.84)^a^	0.002
+ log BNP	0.97 (0.92-1.02)	0.90 (0.84-0.96)^a^	0.72 (0.62-0.85)^a^	<0.001
VE/VCO_2_ slope				
Unadjusted	1.04 (1.02-1.06)^a^	1.05 (1.03-1.07)^a^	1.03 (1.01-1.05)^a^	0.452
MAGGIC risk score	1.03 (1.01-1.05)^a^	1.03 (1.01-1.06)^a^	1.04 (1.01-1.06)^a^	0.637
+ log BNP	1.03 (1.00-1.05)^a^	1.03 (1.01-1.05)^a^	1.03 (1.01-1.06)^a^	0.437

Abbreviations as in Table 1.

a *P* < 0.05.

We also performed 2 sensitivity analyses. First, even when including only patients with peak RER ≥1.05, a higher peak VO_2_ was associated with a lower risk of composite events (Supplemental Table 2). Second, even after adjusting for the modified MAGGIC score excluding the age component, the results remained consistent with those of the primary analysis (Supplemental Table 3).

### Predictive capability of peak VO_2_ or VE/VCO_2_ slope

The added predictive capability of peak VO_2_ or VE/VCO_2_ slope to the MAGGIC risk score, or plus log-transformed BNP for adverse outcomes, was examined using Harrell's C-statistic, NRI, and IDI (Table 3). In patients 20 to 64 years, although adding peak VO_2_ or VE/VCO_2_ slope increased Harrell's C-statistic compared with established risk factors, the differences were not statistically significant. The NRI and IDI did not improve with the addition of peak VO_2_, whereas the addition of VE/VCO_2_ slope showed significant improvement. In patients 65 to 79 years, Harrell's C-statistic of the addition of peak VO_2_ tended to increase, although it was not statistically significant. The NRI and IDI of the added peak VO_2_ were significantly improved. Conversely, the VE/VCO_2_ slope did not show any improvement in predictive capabilities in all analyses. In patients ≥80 years, the addition of VE/VCO_2_ slope showed an increase in Harrell's C-statistic. Although peak VO_2_ tended to improve, it was not statistically significant. The additive value of peak VO_2_ significantly increased the NRI and IDI or VE/VCO_2_ slope to established risk models. Among patients who achieved maximum effort in the sensitivity analyses, the additive value of peak VO_2_ was similar across all age groups (Supplemental Table 4).

**Table 3:** Reclassification Analyses for the Primary Outcome of Peak VO_2_ and VE/VCO_2_ Slope

	Harrell's C-Statistic	*P* Value	NRI	Positive/Negative NRI	*P* Value	IDI	*P* Value
Age ≥80 years							
MAGGIC risk score	0.65 (0.56-0.75)						
MAGGIC risk score + peak VO_2_	0.76 (0.68-0.85)	0.057	0.6661 (0.2568-1.0145)	0.4196/0.2468	0.002	0.1083 (0.0390-0.1776)	0.002
MAGGIC risk score + VE/VCO_2_ slope	0.75 (0.67-0.83)	0.019	0.7063 (0.2966-1.1075)	0.2258/0.4805	<0.001	0.0842 (0.0279-0.1405)	0.003
MAGGIC risk score + log BNP	0.68 (0.59-0.78)						
MAGGIC risk score + log BNP + peak VO_2_	0.77 (0.69-0.85)	0.112	0.6016 (0.1432-0.9795)	0.3548/0.2468	0.005	0.0941 (0.0317-0.1564)	0.003
MAGGIC risk score + log BNP + VE/VCO_2_ slope	0.76 (0.68-0.84)	0.030	0.7708 (0.3512-1.1542)	0.2903/0.4805	<0.001	0.0676 (0.0172-0.1181)	0.008
Age 65–79 years							
MAGGIC risk score	0.71 (0.65-0.77)						
MAGGIC risk score + peak VO_2_	0.74 (0.68-0.79)	0.172	0.3466 (0.0842-0.6137)	0.2836/0.0631	0.013	0.0320 (0.0117-0.0523)	0.002
MAGGIC risk score + VE/VCO_2_ slope	0.71 (0.65-0.77)	0.773	0.2255 (−0.0319 to 0.4844)	−0.0448/0.2703	0.11	0.0152 (−0.0019 to 0.0323)	0.081
MAGGIC risk score + log BNP	0.73 (0.68-0.79)						
MAGGIC risk score + log BNP + peak VO_2_	0.75 (0.69-0.80)	0.446	0.2779 (−0.0130 to 0.5440)	0.2239/0.0541	0.046	0.0250 (0.0070-0.0430)	0.007
MAGGIC risk score + log BNP + VE/VCO_2_ slope	0.73 (0.68-0.79)	0.937	0.2165 (−0.0719 to 0.5140)	−0.0448/0.2613	0.12	0.0125 (−0.0030 to 0.0279)	0.11
Age 20–64 years							
MAGGIC risk score	0.65 (0.59-0.72)						
MAGGIC risk score + peak VO_2_	0.68 (0.61-0.74)	0.051	0.0896 (−0.1924 to 0.3618)	0.1000/-0.0104	0.53	0.0089 (−0.0020 to 0.0198)	0.11
MAGGIC risk score + VE/VCO_2_ slope	0.68 (0.61-0.74)	0.143	0.4482 (0.1662-0.7234)	0.1333/0.3149	0.0016	0.0228 (0.0040-0.0416)	0.017
MAGGIC risk score + log BNP	0.68 (0.62-0.75)						
MAGGIC risk score + log BNP + peak VO_2_	0.69 (0.63-0.76)	0.235	0.1035 (−0.1720 to 0.3945)	0.1000/0.0035	0.47	0.0046 (−0.0035 to 0.0126)	0.27
MAGGIC risk score + log BNP + VE/VCO_2_ slope	0.69 (0.63-0.76)	0.359	0.3790 (0.1010-0.6445)	0.1333/0.2457	0.008	0.0166 (0.0010-0.0322)	0.037

IDI = integrated discrimination index; NRI = net reclassification improvement; other abbreviations as in Table 1.

### Age-stratified time-dependent ROC curve and cutoff value in the aged ≥80 years

**Figure 3:**
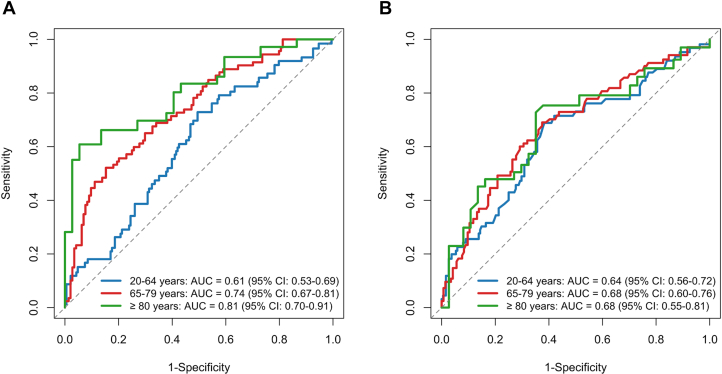
Time-Dependent Receiver-Operating Characteristic Curves of Peak Oxygen Consumption or Minute Ventilation/Carbon Dioxide Production Slope by Age Group Time-dependent receiver-operating characteristic curves are shown for composite adverse outcomes of age-stratified (A) peak VO_2_ and (B) VE/VCO_2_ slope. Peak VO_2_ in patients aged 80 years or older had the highest predictive capabilities, whereas VE/VCO_2_ slope was comparable across age groups. AUC = area under the curve.

Figure 3 shows the time-dependent ROC curve for composite adverse outcome prediction using age-stratified peak VO_2_ and VE/VCO_2_ slope. The AUCs (95% CI) of peak VO_2_ tended to be higher with age (20-64 years: 0.61 [0.53-0.69], 65-79 years: 0.74 [0.67-0.81], ≥80 years: 0.81 [0.70-0.91]), whereas the AUCs (95% CI) of VE/VCO_2_ slope had comparable predictive capabilities across age groups (20-64 years: 0.64 [0.56-0.72], 65-79 years: 0.68 [0.60-0.76], ≥80 years: 0.68 [0.55-0.81]). Furthermore, similar trends in peak VO_2_ for adverse events were observed in patients with peak RER ≥1.05 (Supplemental Figure). In patients ≥80 years, the cutoff values of peak VO_2_ and VE/VCO_2_ slope for adverse events, based on the maximization of the log rank statistic, were estimated as follows: peak VO_2_, 9.6 mL/kg/min (sensitivity 46.9 [29.1-65.3], specificity 92.9 [85.1-97.3], PPV 71.4 [47.8-88.7], and NPV 82.1 [72.9-89.2]); VE/VCO_2_ slope, 37.4 (sensitivity 78.1 [60.0-90.7], specificity 60.7 [49.5-71.2], PPV 43.1 [30.2-56.8], and NPV 87.9 [76.7-95.0]).

## Discussion

In this study, both peak VO_2_ and VE/VCO_2_ slope were consistently associated with adverse outcomes in patients aged ≥80 years. Notably, the prognostic association of peak VO_2_ became stronger with advancing age, and its incremental value over established risk markers appeared to be more robust (Central Illustration). These findings suggest that CPET may play an important role in risk stratification, particularly in the oldest old patients with HF.

Although peak VO_2_ and VE/VCO_2_ slope are established prognostic predictors,^4^^,^^16^, ^17^, ^18^^,^^25^^,^^26^ their evaluation in older HF patients, particularly octogenarians, has been limited. Although cardiorespiratory fitness is a consistent determinant of survival across the lifespan in the general population,^27^ the complex maladaptive responses inherent in HF necessitate integrative physiological assessment via CPET. Addressing the historical focus on middle-aged cohorts, this study demonstrates that CPET parameters remain robust predictors of adverse outcomes across a wide age spectrum, including the oldest old aged 80 years or older. Exclusive to this study, we identified an age-dependent increase in the prognostic power of peak VO_2_, which exhibited its highest predictive capability in patients aged ≥80 years. These findings suggest that, regardless of age, physiological assessment via CPET provides critical prognostic information that extends beyond existing risk models.

In recent years, studies of cardiovascular disease have moved beyond chronological age, emphasizing the importance of the biological and physiological aspects of aging.^28^^,^^29^ A previous study showed that fitness-associated biological age based on metabolic equivalents of tasks was a strong predictor of mortality and myocardial infarction compared to chronological age.^30^ Moreover, a recent report showed that the survival rates of patients aged ≥80 years with the highest fitness level are on par with or exceed those of their 60–70s counterparts with submoderate fitness levels.^31^ This study showed an age-related decline in peak VO_2_ and ventilatory efficiency. In particular, peak VO_2_ and VE/VCO_2_ slope represent a physiological hallmark of aging,^32^, ^33^, ^34^, ^35^ and these associations for adverse composite outcomes were consistently observed across all age groups. These results suggest that assessments based solely on chronological age may provide misleading information and may be inadequate for appropriate patient risk stratification.

Our findings of age-dependent differences may be partly explained by physiological and age-related features. Peak VO_2_ reflects the integrated function of the oxygen transport system, including pulmonary oxygen uptake, cardiac output, and peripheral oxygen utilization. In older patients with HF, particularly those aged ≥80 years, there has been a high prevalence of HFpEF,^36^ as well as geriatric conditions such as sarcopenia, frailty, and malnutrition.^37^^,^^38^ These conditions are closely associated with impaired skeletal muscle function and reduced peripheral oxygen utilization, which are key determinants of exercise capacity.^39^ As these peripheral factors are not fully captured by conventional risk markers such as the MAGGIC risk score or BNP, this may have contributed to peak VO_2_ retaining relatively robust prognostic value in the oldest old age group in this study.

Our findings have important clinical implications. Although chronological age has traditionally been considered in assessing aging, there is a growing recognition that heterogeneity in aging must be considered even within the same chronological age groups.^40^ This highlights the increasing focus on biological rather than chronological age and underscores the need for precise physiological assessment. This study provides insights that may aid in risk stratification of patients with HF, including those aged ≥80 years, and therefore highlights the robust prognostic value of CPET parameters in this population. Importantly, advanced therapies such as heart transplantation or left ventricular assist device implantation are rarely considered in this age group regardless of CPET findings, whereas other interventions, including valvular procedures, cardiac resynchronization therapy, cardiac rehabilitation, and guideline-directed medical therapy, remain clinically relevant^41^^,^^42^ and may be better informed by CPET. The value of CPET in guiding decision-making for invasive therapeutic strategies warrants additional studies. Japan has one of the highest proportions of older adults among developed nations, effectively serving as a forerunner of global population aging. In this context, our findings may provide essential insights into addressing the challenges of forthcoming super-aged societies. However, the robustness of our novel findings requires further investigation.

### Study limitations

Despite its significant contributions, this study has some limitations. First, because this was a multicenter study, the CPET protocol and instrument were not uniform in 8 participating institutions and all the institutions were in Japan. These factors may limit the generalizability of our findings. Nevertheless, all CPET were performed by trained and experienced technicians, which helped minimize measurement errors. In addition, the relatively lower mean body mass index in the present study compared with that reported in U.S. populations with HF may limit the generalizability of our findings to the dominant cardiometabolic HFpEF phenotype observed in the United States. Second, because this was a retrospective observational study, only patients who underwent CPET were included. Selection bias may be present, as patients who were not only ineligible for CPET but also unable to perform it due to aging-related and geriatric diseases (eg, those with severe frailty, cerebrovascular diseases, neurodegenerative diseases, and orthopedic disorders) were not included. Third, the presence of frailty, which is an important prognostic factor for HF, was not considered in this study. However, because CPET was undertaken, it is likely that patients with severe frailty were not included in the study. In clinical practice, patients with severe frailty (e.g., Clinical Frailty Index ≥7) or extremely poor prognosis are often not considered suitable candidates for CPET, as the results are unlikely to alter HF management. Therefore, our findings should be interpreted within the selected population of patients who are deemed capable of undergoing CPET. Finally, due to the constraints of sample size, we were unable to examine the interactions between peak VO_2_ or VE/VCO_2_ slope and other clinical variables in relation to prognosis. In addition, the number of patients aged close to 90 years was relatively small in this study; thus, our findings in this population should be interpreted cautiously. This limitation of sample size may have limited statistical power to detect consistent incremental prognostic value across CPET parameters and age-stratified groups. The variability observed in Harrell's C-statistic, NRI, and IDI may reflect limited power and multiple comparisons rather than a true absence of association. These findings should be interpreted with caution.

## Conclusions

CPET parameters were associated with adverse outcomes across the age spectrum, with peak VO_2_ exhibiting its highest predictive capability in patients aged ≥80 years. This highlights that physiological reserve is a critical determinant of survival in the oldest old, validating CPET as a vital tool for risk stratification in geriatric HF.

Perspectives **COMPETENCY IN MEDICAL KNOWLEDGE:** CPET parameters have critical prognostic information across the age spectrum in patients with HF, with the prognostic value of peak VO_2_ progressively increasing with advancing age and exhibiting its strongest predictive capability in patients aged ≥80 years. These parameters provided incremental prognostic value beyond the MAGGIC risk score and BNP.

**TRANSLATIONAL OUTLOOK:** The novel finding that age-dependent differences in the prognostic contribution, especially that of peak VO_2_, warrant further investigation worldwide across patients with geriatric diseases, including frailty, to validate CPET as a vital tool in geriatric HF.

**Central Illustration:**
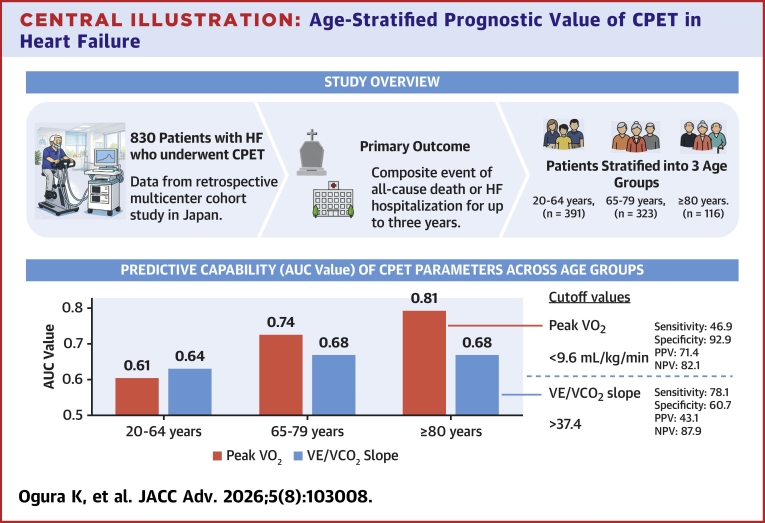
Age-Stratified Prognostic Value of CPET in Heart Failure In patients with heart failure, the prognostic value of peak VO_2_ progressively increased with advancing age and was highest in those aged 80 years or older, whereas VE/VCO_2_ slope showed comparable prognostic value across age groups. AUC = area under the curve; CPET = cardiopulmonary exercise testing; HF = heart failure; VE/VCO_2_ = minute ventilation/carbon dioxide production; VO_2_ = oxygen consumption.

## Funding support and author disclosures

This study was partially supported by JSPS KAKENHI (grant numbers 26K02755). Dr Matsue received honoraria from Otsuka Pharmaceutical Co., Ltd., EN Otsuka Pharmaceutical Co., Ltd., Novartis Pharma K.K., Ono Pharmaceutical Co., Ltd., AstraZeneca K.K., Nippon Boehringer Ingelheim Co., Ltd., Bayer Yakuhin, Ltd., Kyowa Kirin Co., Ltd., Pfizer Japan Inc., and Alnylam Japan K.K; and he also received collaborative research funding from Otsuka Pharmaceutical Co., Ltd., EN Otsuka Pharmaceutical Co., Ltd., Bayer Yakuhin, Ltd., Pfizer Japan Inc., Nippon Boehringer Ingelheim Co., Ltd., AstraZeneca K.K., Roche Diagnostics K.K., Grace Imaging Inc., and Omron Healthcare Co., Ltd. All other authors have reported that they have no relationships relevant to the contents of this paper to disclose.
