# Supplementary material for: Age-Stratified Prognostic Value of Cardiopulmonary Exercise Testing Parameters in Patients With Heart Failure
Source: JACC Adv. 2026 Jul 15;5(8):103008. doi: 10.1016/j.jacadv.2026.103008 (PMC13384006; doi:10.1016/j.jacadv.2026.103008)
Supplement: Supplemental Material [file mmc1.pdf]

Supplemental Table 1. Age-specific mean values of Peak VO_2.

	Male		Female	
Age, y	n	Mean ± SD	n	Mean ± SD
20–29	9	20.5 ± 6.0	10	19.0 ± 4.4
30–39	34	18.9 ± 7.8	16	14.3 ± 4.3
40–49	98	18.3 ± 5.4	27	15.0 ± 4.7
50–59	114	18.8 ± 5.3	22	15.5 ± 5.0
60–69	108	16.2 ± 4.7	50	14.9 ± 9.4
70–79	149	15.1 ± 4.7	77	14.6 ± 4.7
≥ 80	79	12.9 ± 3.8	37	12.0 ± 3.2

Peak VO_2, mL/kg/min.

Supplemental Table 2. Cox regression analyses for peak VO_2 as predictors in patients with peak RER ≥ 1.05.

	20–64 years	65–79 years	≥ 80 years	
No. of events, n/N (%)	63/343 (18.4)	60/255 (23.5)	20/75 (26.7)	
	HR (95% CI)	HR (95% CI)	HR (95% CI)	*P* for interaction
Unadjusted	0.92 (0.87–0.98)*	0.85 (0.80–0.91)*	0.67 (0.56–0.81)*	0.003
MAGGIC risk score	0.96 (0.90–1.02)	0.89 (0.83–0.96)*	0.71 (0.59–0.86)*	0.012
+ log BNP	0.97 (0.91–1.03)	0.91 (0.84–0.98)*	0.71 (0.58–0.86)*	0.008

* $P < 0.05$

BNP = brain natriuretic peptide; HR = Hazard ratio; MAGGIC = Meta-analysis Global Group in Chronic Heart Failure; VO_2 = oxygen consumption.

Supplemental Table 3. Cox regression analyses for peak VO_2 and VE/VCO_2 slope adjusted the modified MAGGIC score.

	20–64 years	65–79 years	≥ 80 years	
Peak VO_2	HR (95% CI)	HR (95% CI)	HR (95% CI)	*P* for interaction
Modified MAGGIC risk score	0.95 (0.90–1.01)	0.88 (0.83–0.94)*	0.72 (0.61–0.84)*	<0.001
+ log BNP	0.97 (0.91–1.02)	0.90 (0.84–0.96)*	0.72 (0.61–0.84)*	<0.001
VE/VCO_2 slope	HR (95% CI)	HR (95% CI)	HR (95% CI)	*P* for interaction
Modified MAGGIC risk score	1.03 (1.01–1.05)*	1.03 (1.01–1.05)*	1.04 (1.01–1.06)*	0.555
+ log BNP	1.03 (1.00–1.05)*	1.03 (1.01–1.05)*	1.04 (1.01–1.06)*	0.384

* $P < 0.05$; adjusted the modified MAGGIC risk score excluding the age component.

BNP = brain natriuretic peptide; HR = Hazard ratio; MAGGIC = Meta-analysis Global Group in Chronic Heart Failure; VE/VCO_2 = minute ventilation/carbon dioxide production; VO_2 = oxygen consumption.

Supplemental Table 4. Reclassification analyses for the primary outcome of peak VO_2 in patients with peak RER ≥ 1.05.

	Harrell's C- Statistic	*P* value	NRI	Positive / Negative NRI	*P* value	IDI	*P* value
≥ 80 years							
MAGGIC risk score	0.70 (0.68–0.89)						
MAGGIC risk score + peak VO_2	0.79 (0.68–0.89)	0.253	0.7136 (0.1666–1.1845)	0.4737 / 0.2400	0.015	0.1060 (0.0088–0.2033)	0.03
MAGGIC risk score + log BNP	0.71 (0.59–0.84)						
MAGGIC risk score + log BNP + peak VO_2	0.79 (0.68–0.89)	0.329	0.7284 (0.2224–1.2009)	0.3684 / 0.3600	0.009	0.1094 (0.0148–0.2039)	0.02
65–79 years							
MAGGIC risk score	0.72 (0.66–0.79)						
MAGGIC risk score + peak VO_2	0.74 (0.68–0.79)	0.490	0.3255 (0.0237–0.6128)	0.2157 / 0.1098	0.04	0.0261 (0.0050–0.0473)	0.02
MAGGIC risk score + log BNP	0.74 (0.67–0.80)						
MAGGIC risk score + log BNP + peak VO_2	0.74 (0.68–0.81)	0.731	0.3140 (0.0151–0.6124)	0.2157 / 0.0983	0.049	0.0220 (0.0024–0.0416)	0.03
20–64 years							
MAGGIC risk score	0.65 (0.58–0.72)						
MAGGIC risk score + peak VO_2	0.67 (0.60–0.74)	0.093	0.0308 (-0.2536–0.3126)	0.0714 / -0.0407	0.84	0.0063 (-0.0031–0.0157)	0.19

MAGGIC risk score + log BNP	0.68 (0.61–0.75)						
MAGGIC risk score + log BNP + peak VO_2	0.69 (0.62–0.75)	0.360	0.0665 (-0.2099–0.3501)	0.1071 / -0.0407	0.65	0.0030 (-0.0035–0.0095)	0.37

BNP = brain natriuretic peptide; IDI = integrated discrimination index; MAGGIC = Meta-analysis Global Group in Chronic Heart Failure; NRI = net reclassification improvement; VE/VCO_2 = minute ventilation/carbon dioxide production; VO_2 = oxygen consumption.

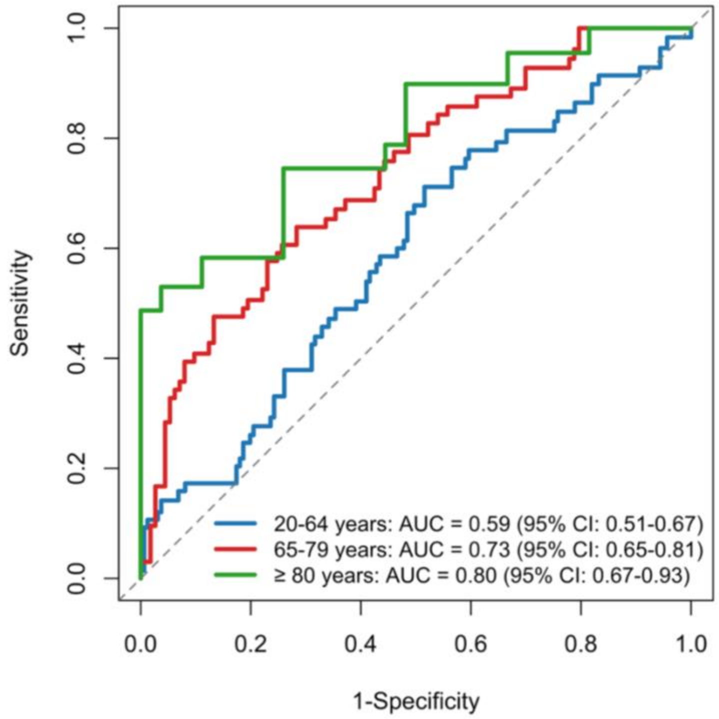

Supplemental Figure. Time-dependent receiver-operating characteristics curve analyses of peak VO_2 in patients with peak RER ≥ 1.05.

Time-dependent receiver-operating characteristic curves for composite adverse outcomes demonstrated that peak VO_2 in patients aged 80 years or older consistently showed the highest predictive capabilities, even in patients with peak RER ≥ 1.05.

AUC: area under the curve.
